# Supplementary material for: Venomix: a simple bioinformatic pipeline for identifying and characterizing toxin gene candidates from transcriptomic data
Source: PeerJ. 2018 Jul 31;6:e5361. doi: 10.7717/peerj.5361 (PMC6074769; doi:10.7717/peerj.5361)
Supplement: Supplemental Information 6 [file peerj-06-5361-s006.gz › FinalOutput_1E-20/Astacin-like_metalloprotease_toxin_1_1/finaltree.pdf]

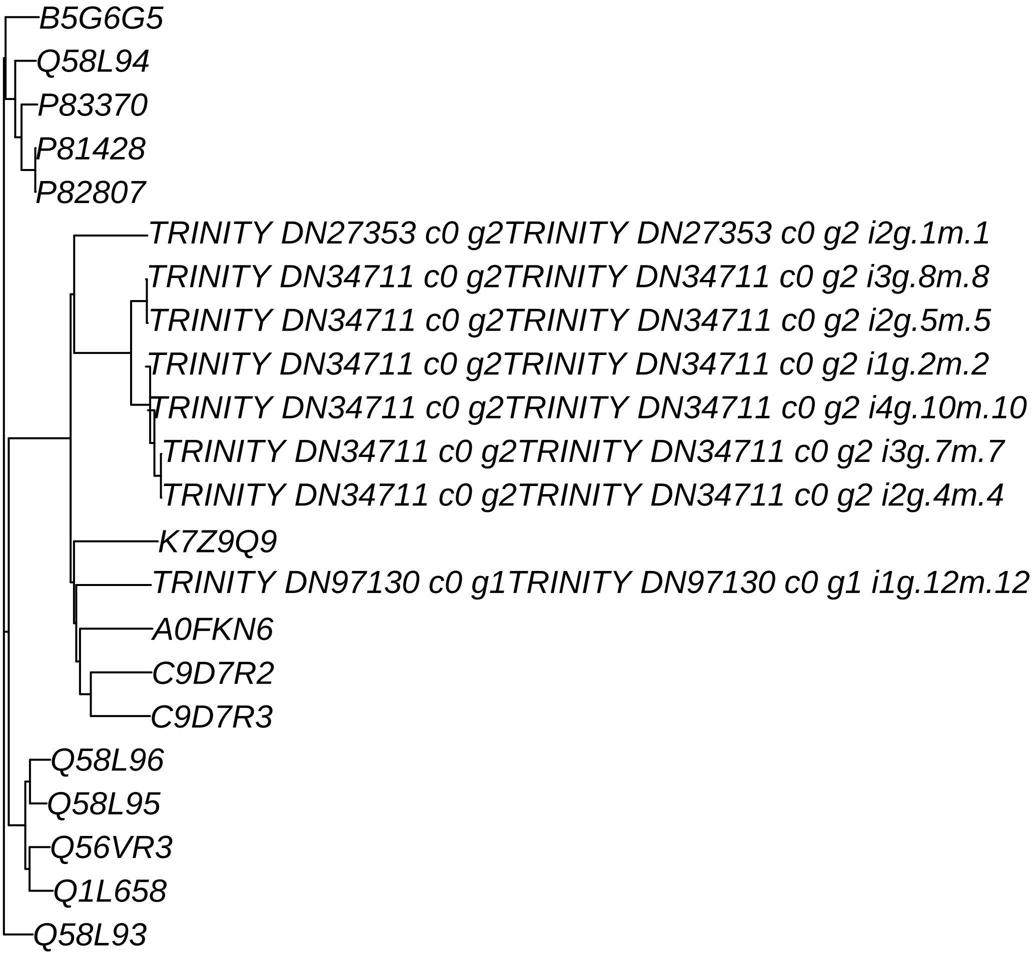
B5G6G5
Q58L94
P83370
P81428
P82807
TRINITY DN27353 c0 g2TRINITY DN27353 c0 g2 i2g.1m.1
TRINITY DN34711 c0 g2TRINITY DN34711 c0 g2 i3g.8m.8
TRINITY DN34711 c0 g2TRINITY DN34711 c0 g2 i2g.5m.5
TRINITY DN34711 c0 g2TRINITY DN34711 c0 g2 i1g.2m.2
TRINITY DN34711 c0 g2TRINITY DN34711 c0 g2 i4g.10m.10
TRINITY DN34711 c0 g2TRINITY DN34711 c0 g2 i3g.7m.7
TRINITY DN34711 c0 g2TRINITY DN34711 c0 g2 i2g.4m.4
K7Z9Q9
TRINITY DN97130 c0 g1TRINITY DN97130 c0 g1 i1g.12m.12
A0FKN6
C9D7R2
C9D7R3
Q58L96
Q58L95
Q56VR3
Q1L658
Q58L93
